# On the dynamics of the human endocrine pancreas and potential consequences for the development of type 1 diabetes

**DOI:** 10.1007/s00592-019-01420-8

**Published:** 2019-09-13

**Authors:** Oskar Skog, Olle Korsgren

**Affiliations:** 1 Department of Immunology, Genetics and Pathology, Uppsala University, Uppsala, Sweden; 2 Department of Clinical Chemistry and Transfusion Medicine, Institute of Biomedicine, University of Gothenburg, Gothenburg, Sweden

**Keywords:** Type 1 diabetes, Hyperemia, Beta-cell turnover, Islet endothelial cells, Proliferation

## Abstract

Little is known about the human islet life span, and beta-cell neogenesis is generally considered rare in adults. However, based on available data on beta-cell proliferation, calculations can be made suggesting that the dynamics of the endocrine pancreas is considerable even during adulthood, with islet neogenesis and a sustained increase in size of already formed islets. Islet-associated hemorrhages, frequently observed in most mammals including humans, could account for a considerable loss of islet parenchyma balancing the constant beta-cell proliferation. Notably, in subjects with type 1 diabetes, periductal accumulation of leukocytes and fibrosis is frequently observed, findings that are likely to negatively affect islet neogenesis from endocrine progenitor cells present in the periductal area. Impaired neogenesis would disrupt the balance, result in loss of islet mass, and eventually lead to beta-cell deficiency and compromised glucose metabolism, with increased islet workload and blood perfusion of remaining islets. These changes would impose initiation of a vicious circle further increasing the frequency of vascular events and hemorrhages within remaining islets until the patient eventually loses all beta-cells and becomes c-peptide negative.

## The turnover of beta-cells

Little is known concerning the lifespan of the human beta-cell. The formation of lipofuscin bodies (non-degradable lipids and proteins in the lysosomes) has been utilized as a tool to assess the longevity of cells. In proliferating human insulinoma cells, the lipofuscin content is low and unrelated to the age of the affected subject. However, only a low proportion of human beta-cells has been reported to lack lipofuscin bodies, even in very young children, i.e., only 11% in 1-year-olds and 5% in 5-year-olds. In adults, the percentage of beta-cells without lipofuscin bodies is 2–3%. The accumulation of lipofuscin in almost all beta-cells has been interpreted to show longevity of beta-cells and that beta-cell proliferation, or neogenesis from endocrine precursors, is uncommon in the typical human pancreas [1]. However, accumulation of lipofuscin bodies is not solely dependent on the rate of cell proliferation and dilution of non-degradable waste products, but also on the metabolic activity of the cell [2]. The insulin-producing beta-cells are among the most metabolically active cells in our bodies, and the results of Cnop and colleagues [1], indicating that an absolute majority of the beta-cells in 1-year-old children already contain lipofuscin bodies and strongly suggest that even relatively newly formed beta-cells rapidly accumulate lipofuscin bodies and that the presence of lipofuscin in beta-cells constitutes a sign of cellular senescence that should be interpreted with caution. Perhaps, the most interesting observation is that even in adults, 2–3% of beta-cells do not contain lipofuscin bodies [1], implying that these cells represent either an actively proliferating pool of beta-cells or that they have been recently generated through a process of differentiation from an endocrine precursor.

Human beta-cell proliferation is reportedly low. However, the most commonly used proliferation marker, Ki67, has been shown to underestimate the rate of islet cell proliferation in autopsy specimens by a factor of 3–6 due to autolysis [3]. The range of beta-cell proliferation in surgical biopsies or after transplantation of human islets to immunoincompetent mice has been reported to be 0.1–0.7% [3–6]. Markedly increased beta-cell replication in subjects with recent-onset T1D has been reported by some investigators [7, 8], but not by others [9]. Nonetheless, cell proliferation seems not to be uniform among beta-cells, and the role of several novel heterogeneity markers for beta-cell maturation, function, and proliferation has recently been elegantly reviewed [10]. Expression of *flattop* (Fltp), a Wnt/planar cell polarity effector and reporter gene, can be used to distinguish proliferation competent from non-replicating β-cells [11]. Since the duration of the cell cycle in most non-malignant cells is about 24 h, the fraction of Ki67-positive cells at a given time point shows the fraction of time that each cell spends in the cycling rather than in the non-cycling (*G*_0_) state. With this assumption, the mean beta-cell birth rate (%/year) can be calculated. Also, assuming a maintained total beta-cell volume in adults, the corresponding mean beta-cell lifespan can be calculated. Calculating with the lower range of the figures provided in the literature for human beta-cell proliferation [3–6, 12], a labeling index (LI) of 0.1% tells us that out of 1000 beta-cells only 1 cell divides per day. This is equivalent of 365 dividing beta-cells per thousand cells per year, corresponding to a doubling of the beta-cells within a 3-year period. Assuming an LI of 0.4% in beta-cells, a doubling of the beta-cell mass occurs in less than 1 year [3]. These astonishing results may in fact even represent an underestimate of the total expansion of the beta-cells, since neogenesis of new beta-cells from the ductal epithelium, as elegantly shown in rodents, is not considered [13]. This type of mechanistic studies cannot, for obvious reasons, be conducted in humans. However, several morphological studies of the human pancreas in lean, obese, and type 2 diabetic subjects, as well during pregnancy, strongly support a similar process to be of importance to maintain and increase beta-cell mass in man [14–17].

To prevent an expansion of the beta-cell mass over the human lifetime, a balancing loss of beta-cells must occur. However, human beta-cell apoptosis seems an even more rare event. In an extensive study of autopsy specimens, only nine events were observed in 236,771 cells examined in a total of five subjects [17], calling for hereto-unidentified mechanisms of beta-cell death.

## The vascularity and blood perfusion of the islets

To maintain the high metabolic activity of the islet cells, and the ability of beta-cells to sense glucose and secrete insulin, the islets (that constitute only 1–2% of the total pancreatic volume) under normoglycemic conditions receive about 10% of the total pancreatic blood flow, as shown in rats [18]. Under hyperglycemic conditions, blood perfusion in the islets is rapidly doubled, mainly via an activation of the vagal nerve, and subsequently maintained at this high level by the local release of metabolic mediators, e.g., adenosine, until normoglycemia ensures [18]. This massive increase in blood perfusion results in a doubling of the intra-islet capillary pressure. Also, the capillary endothelium of the islets is optimized to facilitate the glucose-sensing and insulin secretion capacity of the beta-cells. It appears that almost every beta-cell is in direct contact with a capillary. The intra-islet endothelial cells are slender, with numerous fenestrae, and separated from the endocrine cells only by a double basal membrane.

Similar to the brain, the islets of Langerhans show a pronounced deficiency of lymphatic capillaries [19], a finding with implications for the regulation of interstitial fluid transport in the endocrine pancreas. In most organs, extracellular interstitial fluid is continuously formed by filtration from blood capillaries, collected by lymphatic capillaries, and finally recirculated back to the blood via the thoracic duct. During periods of increased insulin resistance, e.g., infections and obesity, or of manifest hyperglycemia, the increased blood perfusion in the absence of lymphatic capillaries would result in increased formation of extracellular fluids and therefore an increased interstitial pressure within the islets. The extracellular volume in human islets under resting conditions has been estimated to be about 14% of the total islet volume [20], and a fivefold increase in the accumulation of hyaluronan and hyaladherins in the pericapillary space between the two basal membranes in insulin-containing islets has been reported in subjects with recent-onset T1D when compared to non-diabetic controls [21]. The lack of an efficient transportation system of accumulated extracellular fluids via a lymphatic capillary network increases the intra-islet capillary pressure and cause augmented tension on the endothelial cell lining of the blood capillaries further increasing the risk for microvascular bleedings.

## The vascular bed of the islets as a hereto-neglected “locus minoris resistentiae”

**Fig. 1:**
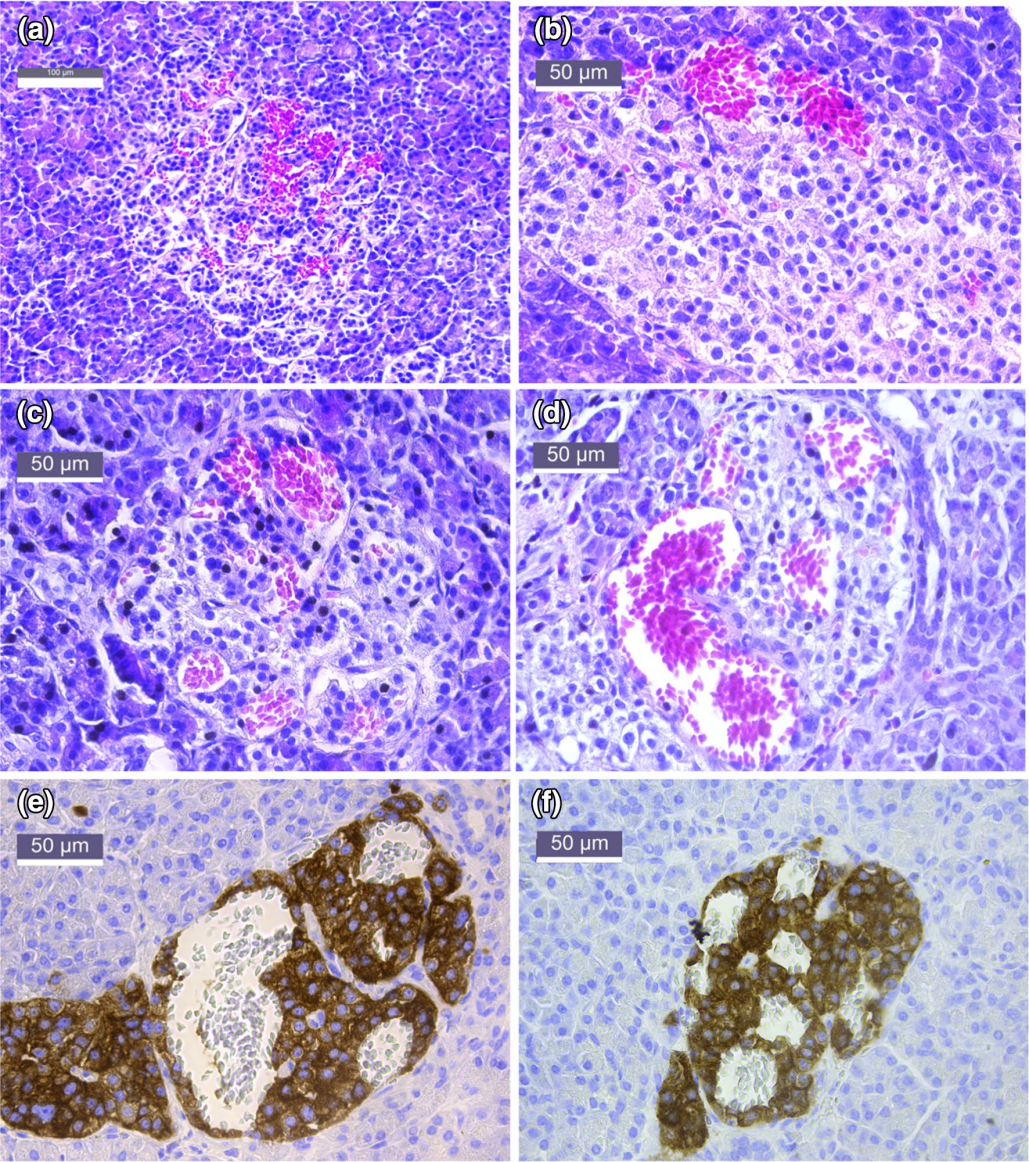
Islets with hemorrhages in organ donors without any previously known pancreatic disease or diabetes. Islets from these donors were successfully isolated and released for clinical transplantation. Sections were stained with hematoxylin and eosin in **a**–**d**, making erythrocytes stain bright pink, and with hematoxylin only in **e** and **f**, making erythrocytes appear gray. In **e** and **f**, immunohistochemical staining for synaptophysin (brown) was used to visualize islet tissue. Original magnification is × 20 in A and × 40 in **b**–**f**; black bar represents 100 µm in **a** and 50 µm in **b**–**f** (color figure online)

A frequent finding in the pancreas of most species is that of so-called hyperemic or hemorrhagic islets [22–25] (Fig. 1), i.e., microvascular bleedings within the islet parenchyma. This type of vascular catastrophes constitutes the underlying mechanism for the spontaneous development of diabetes in male Torii rats [26]. At 8–10 week of age, the pancreatic islets show congestion of blood and hemorrhages, and their beta-cell volume decreases. At this prediabetic stage, animals remain normoglycemic but have significantly lower fasting plasma insulin levels than control rats. Islet immune cell infiltration and fibrosis are seen within and around 3.2% of all the islets at 10 weeks and in almost all the islets at 20 weeks. At 14 weeks when the rats become glucose-intolerant but yet not overtly diabetic, beta-cells significantly decrease in numbers. When overt diabetes develops in this model, almost all of the beta-cells are lost from the islets, whereas other endocrine cells such as glucagon, somatostatin, or PP-containing cells are still present, although reduced in numbers. At 24 weeks, the rats show overt hyperglycemia (> 25 mmol/L) and marked hypoinsulinemia accompanied by decreased body weight and BMI, features characteristic of human T1D [26].

In non-diabetic humans, hemorrhagic islets have been reported in half of all pancreases examined and then in 3–4% of all islets, irrespective of whether pancreatic biopsies were obtained from organ donors or from areas with “ normal” pancreatic tissue in samples excised at elective surgical procedures (e.g., after pancreatoduodenectomy for pancreatic carcinoma) during surgery [24]. A slight increase in frequency of hemorrhagic islets was observed in donors with their last recorded systolic blood pressure above 140 mm Hg and/or diastolic blood pressure above 90 mm Hg; however, the majority of organ donors with hemorrhagic islets had normal blood pressure. Notably, donation after brain death or donation after cardiac death had no effect on the presence of hemorrhagic islets. However, there was a slight increase in the frequency of donors with hemorrhagic islets correlated with time in the intensive care unit (3.4 days in donors with hemorrhagic islets compared with 2.2 days in donors without hemorrhagic islets). Importantly, in a large animal model in pigs, where all these confounding factors can be standardized, hemorrhagic islets were found in 48% of the purebred and in 68% of the crossbred pigs. Notably, the reported frequency is likely an underestimation of the true value since only a minor part of the pancreas volume was examined. Similar to the human pancreas, 3.3 ± 3.1%, and 3.1 ± 4.7% of all assessed islets in these pancreases were hemorrhagic in purebred and crossbred pigs, respectively [23].

The extensive delicate and fenestrated capillary network within the islets requires regular maintenance to sustain their high and dynamic blood perfusion. Platelets are not only required for hemostasis but also for their capacity to maintain the structural integrity of capillaries and postcapillary venules [27]. Platelets support the resting vascular endothelium by (1) physically blocking gaps in the vascular lining, (2) promoting the growth of endothelial cells (3) maintaining the endothelial ultrastructure, and (4) enhancing the barrier function of the endothelium by releasing soluble factors. In experimental studies, platelets seem to be critical for preventing bleeding at sites of local inflammation [28]. This effect appears to be independent of GPIIb-/IIIa-mediated aggregation but dependent on GPVI and CLEC-2, the only immunoreceptor tyrosine-based activation motif (ITAM) receptor present on mouse platelets (human platelets further express the IgG Fc receptor FcgRIIA). Platelets treated with inhibitors of the spleen tyrosine kinase (Syk) and Bruton’s tyrosine kinase, transducers of GPVI and CLEC-2 signaling, show an impaired ability to prevent bleeding without affecting their recruitment to the site of inflammation. Syk plays an essential role in the formation of platelet filopodia, but not in GPVI potentiation of platelet activation by thrombin.

Changes in platelet shape and platelet secretion can be triggered independently of aggregation, notably in response to collagen. In particular, platelets pretreated with thrombin and emptied of their contents before transfusion to thrombocytopenic mice fail to prevent bleedings, suggesting a potential role for paracrine interactions between platelets and endothelial cells that is potentially mediated by the bioactive endothelial barrier-protective lipid sphingosine-1-phosphate (S1P) [28]. Promotion of vascular integrity by S1P is dependent on expression of the S1P receptor 1 on endothelial cells [29]. S1P receptor 1 is expressed on the endothelial cells of the islets and is increased by short-term but markedly decreased by long-term hyperglycemia [30]. S1P activation of the S1P1 receptor functions in an anti-inflammatory manner and prevents monocyte adherence to the vascular endothelium. Notably, aortas from diabetic NOD mice bind sevenfold more monocytes than non-diabetic littermates and pretreatment of the aortas with S1P almost totally abrogated the accumulation of monocytes [31]. Erythrocytes and endothelial cells seem to be the main sources of S1P in plasma; however, platelets also express abundant amounts of S1P [32], and during inflammation and vascular damage, platelets become activated and locally release high levels of S1P [32]. It is noteworthy that these platelet-mediated endothelial protective processes are inhibited by aspirin and NSAIDs, often used to relieve symptoms of infections [33–36]. Especially worrisome in this regard is the fact that the increase in T1D incidence is paralleled by the increased use of these medications and the observation that the use of NSAIDs is especially frequent in countries with a high incidence of T1D, such as Sweden and Finland [37–39].

In line with an important role for platelets in maintaining the integrity of islet capillaries, hemorrhagic islets and infiltration of mononuclear cells are observed already after 12 h in non-diabetic rats with severe thrombocytopenia induced by anti-platelet serum [40]. Supporting the dependence on ITAM for the protection of islet vascular integrity, treatment of rats with specific inhibitors of Syk and Bruton’s tyrosine kinases causes distinct pancreatic lesions characterized by multifocal intra- and peri-islet hemorrhage, inflammation, and fibrosis [41, 42]. In animals on maintenance treatment with these specific Syk inhibitors, glucose metabolism starts to deteriorate after only 14 days [42]. The sensitivity of rats to Syk and Bruton’s tyrosine kinase inhibitors seems to be strain dependent and to correlate with the background rate of spontaneous hemorrhagic islets [41, 43].

## The turnover of islets

Carefully performed morphometric studies by Ogilvie as early as the 1930s have shown that during the first years of life, the human islet tissue increases more as the result of a gain in islet number than an increase in size within individual islets [44]. The number of islets seems to be established around the age of about 3 years, and from this age, the marked increase in islet mass during childhood and adolescence seems to be mainly due to enlargement of the average islet size. After 20 years of age, the islet mass becomes stabilized in terms of both the total number of islets and the islet size distribution.

These early results concerning the endocrine human pancreas demonstrate the dynamics of both the formation of new islets and the expansion of small islets into large islets. At present, a more static situation is often depicted, with a huge number of small islets with a diameter of 50 µm or less and only very few islets with a diameter above 250 µm; even so, the most important contribution to the total islet volume is by the medium-sized islets of 100–150 µm in diameter [45]. This stationary view of the islet compartment has dominated in recent years, and the dynamics of the endocrine pancreas seems underestimated.

The detailed dynamics of human islets are not known, but one can envision that all islets have eventually been formed as small clusters of endocrine cells. As these cells slowly replicate, the islets grow in size. If we assume an LI in the lower range of those reported (0.1%) [3–6, 12], an islet with a diameter of 50 µm will increase eight times in volume and turn into an islet with a diameter of 100 µm over a period of 9 years; with an LI close to the average of that reported in the literature (0.4%) [3–6, 12], this growth of an islet would be accomplished in only 3 years. Taking into consideration also the process of neoformation of beta-cells, the growth of the islets would occur at an even faster rate [13].

A stationary number of islets with even a low beta-cell proliferation rate of 0.1% would, over the lifetime of a human being, result in the accumulation of very large islets and no remaining small islets. Since islet size distribution remains unchanged from adolescence to the upper limit of the human lifespan, other processes must be in operation: The presence of numerous small islets implies constant islet neogenesis, and the presence of only a few large islets implies that entire islets are sometimes lost during their progression into large islets. The specific location in the pancreas likely governs both the growth rate as well as the maximal islet size; only when positioned in a location providing optimal support in terms of blood supply and innervation can an islet reach its maximal size of about 250 µm in diameter.

Hemorrhagic islets have been reported to represent 3–4% of all islets in about 50% of subjects examined [24]. Assuming that (1) on average, the percentage of islets with a hemorrhage is about 1% (2) that this vascular catastrophe causes a 50% loss of islet parenchyma in affected islets, and (3) bleeding within an islet is resorbed within a week, several interesting calculations can be made concerning the dynamics of the islets in humans. With the stated assumptions, these islet lesions would cause a loss of islet parenchyma of 0.5% per week, equivalent to a 25% loss per year. Notably, this tentative figure fits remarkable well with the estimated renewal of beta-cells if one assumes an LI of only about 0.1% [3–6, 12].

Also, assuming that a hemorrhagic episode would cause loss of the entire islets in about 25% of occasions 0.25% of all islets would be lost during 1 week, corresponding to a loss of 12.5% of all islets over 1 year. With a constant total number of islets in humans in the range of 1–2 million, this would mean a loss of about 125,000–250,000 islets per year, a figure that must be balanced by the formation of a similar number of new islets [15, 17, 44]. Again, this tentative figure fits remarkably well with a beta-cell LI of 0.1% [3–6, 12] and a maintained size distribution of islets [45, 46].

## Clinical implications of lost vascular integrity in the islets

Progression to overt T1D occurs only slowly over many years; 10 years after seroconversion, about 70% of children with high-risk HLA alleles and multiple islet autoantibodies have been diagnosed with T1D [47]. Thus, even if the clinical onset of T1D is usually abrupt, the injurious processes that eventually cause the disease seem to have been in place for many years. Also, there is only a gradual loss of the remaining insulin-producing cells after T1D diagnosis, as evidenced by a decline in c-peptide over a period of several years, or even decades, in most subjects [48, 49]. Importantly, the long-lasting beta-cells remain functional even several decades after diagnosis of T1D, as evidenced by increased secretion of c-peptide in response to a mixed meal.

Focal lesions of acute pancreatitis and an accumulation of leukocytes, often around the ducts, are frequently observed in subjects with recent-onset (T1D) [50, 51], and most T1D patients display extensive periductal fibrosis, the end stage of inflammation [52]. An injurious inflammatory adverse event occurring within the periductal area may reduce the capacity for islet neogenesis, depending on negative implications for stem cells that reside within or adjacent to the ductal epithelium [53, 54]. The number of islets per exocrine tissue (the islet density) is markedly reduced in subjects with T1D when compared to matched non-diabetic subjects [55]. Impaired islet neogenesis would lead to prolonged periods of impaired glucose metabolism and thereby an extensive workload on persisting beta-cells and an accompanying hyperperfusion of blood in remaining islets. In children, these insults would be further aggravated by the physiological increase in insulin demands resulting from rapid body growth and puberty.

In experimental studies of increased work-load in the islets, e.g., during periods of increased insulin resistance, the islet vessel density decreases, and the intra-islet vasculature becomes markedly dilated [56]. Corticosteriods usually induce vasoconstriction and reduced vascular leakage; however, in mice treated for 3 weeks with corticosteroids to induce increased islet workload by increasing insulin resistance, micro-hemorrhages were observed within the islets [57]. Similarly, vascular congestion and hemorrhages in islets, accompanied by mononuclear cell and T-lymphocytic infiltration, have been reported in several spontaneous rat models of increased insulin resistance [58, 59].

A frequent finding in the pancreas of most species is that of so-called hyperemic or hemorrhagic islets [22–25]. Also, in humans hemorrhagic events in the islets are seemingly not rare events [24], and it is speculated that this can indeed be regarded as a part of the natural turnover of islets over time in most species. The high level of constitutive expression of tissue factor in the endocrine cells of the islets may find its physiological role as an efficient mechanism for controlling these vascular catastrophic events by instantly helping to demarcate and prevent excessive bleeding within the pancreas [60]. TF is constitutively expressed by cells in the adventitia of the blood vessels and is found in other highly vascularized tissues such as the cerebral cortex, renal glomeruli, and lungs to ascertain instant activation of the extrinsic pathway of coagulation whenever a bleeding occurs [61]. Thrombocytopenia in humans does not regularly result in islet hemorrhages and clinical diabetes. However, during periods of increased islet workload and blood perfusion, thrombocytopenia can result in massive and seemingly specific loss of islets as a result of islet hemorrhages [62–66].

Hemolytic uremic syndrome (HUS) is characterized by acute hemolytic anemia, thrombocytopenia, endothelial cell damage and renal failure. However, HUS is also associated with an increased risk to develop diabetes. The incidence of diabetes in surviving subjects was in a meta-analysis reported to be 3.2%, i.e., a 100-fold increase compared with the general population [67]. Autopsy studies have demonstrated hemorrhages and thrombosis in the islets of Langerhans with preservation of the exocrine pancreas [63, 66]. In other subjects, however, hemorrhagic necrosis of the exocrine pancreas (pancreatitis) is also found. These observations align with the increased risk of islet hemorrhages during periods of severe thrombocytopenia and insulin resistance.

During the period from 1990 to 2010, a rapid increase in the incidence of T1D occurred in Europe, which subsequently seems to have plateaued. This dramatic change in incidence over a short period of time has been interpreted as supporting a decisive role for an infectious environmental agent in the etiology of T1D. Alternatively, the precipitating factor could be the observed increase and subsequently plateauing in body weight in children during the same period of time [68–71]. Fasting blood glucose and HbA1c have, however, not increased in non-diabetic children during this period because of a marked increase in plasma insulin levels, that is, a persistently increased beta-cell workload seems to be required to maintain normoglycemia in children today because of their increase in body weight [72]. Interestingly, higher birth weight and increased weight gain during the first years of life enhance the risk for development of T1D later in life [73, 74]. Also, an often-noted clinical observation in subjects with T1D is the temporary increase in needs of exogenous insulin during infections, which is attributed to increased insulin resistance. Notably, if this occurs in subjects who have recently been diagnosed with T1D with low requirement for exogenous insulin because of preserved c-peptide secretion (during the honeymoon period), the need for insulin after the infection has resolved is often permanently increased, pointing to a loss of beta-cell function in conjunction with the infection. Similarly, insulin resistance accelerates progression to T1D in islet autoantibody-positive relatives in whom insulin secretion is reduced but does not affect progression when insulin secretion is relatively well preserved [75–77]. Collectively, these clinical observations could tentatively be explained by loss of remaining beta-cells because of increased islet workload with concomitant increase in islet blood perfusion resulting in a higher frequency of islet hemorrhages.

Available clinical observations in T1D suggest a mild disease process occurring over several years or even decades. This should be viewed in relation to the total beta-cell mass of only 0.2–1.5 g in non-diabetic adults [17, 78]. To be in line with the slow clinical progress of T1D, the beta-cell-damaging process(es) occurring over many years must be of low intensity or must target, during each episode, only a minor fraction of the beta-cells. The model presented herein with impaired islet neogenesis and loss of islets in hemorrhagic episodes fits well with these clinical observations. Minor hemorrhages are preferentially located in the peri-islet area and, when resolved, explain the often-described peri-insulitis, preferentially consisting of CD8 + tissue-resident memory T cells [79], and the formation of islet autoantibodies as a result of the necrosis of insulin-producing cells in an inflamed microenvironment. Impaired islet neogenesis would eventually lead to a deficiency of beta-cells and compromised glucose metabolism, with increased islet work-load and blood perfusion of the remaining islets. These changes would impose initiation of a vicious circle further increasing the risks of vascular events and hemorrhages within remaining islets until the patient eventually becomes c-peptide negative.
